# Conservation and Sex-Specific Splicing of the *transformer* Gene in the Calliphorids *Cochliomyia hominivorax*, *Cochliomyia macellaria* and *Lucilia sericata*

**DOI:** 10.1371/journal.pone.0056303

**Published:** 2013-02-07

**Authors:** Fang Li, Steven P. Vensko, Esther J. Belikoff, Maxwell J. Scott

**Affiliations:** 1 Department of Genetics, North Carolina State University, Raleigh, North Carolina, United States of America; 2 Department of Entomology, North Carolina State University, Raleigh, North Carolina, United States of America; King’s College London, United Kingdom

## Abstract

Transformer (TRA) promotes female development in several dipteran species including the Australian sheep blowfly *Lucilia cuprina*, the Mediterranean fruit fly, housefly and *Drosophila melanogaster*. *tra* transcripts are sex-specifically spliced such that only the female form encodes full length functional protein. The presence of six predicted TRA/TRA2 binding sites in the sex-specific female intron of the *L. cuprina* gene suggested that *tra* splicing is auto-regulated as in medfly and housefly. With the aim of identifying conserved motifs that may play a role in *tra* sex-specific splicing, here we have isolated and characterized the *tra* gene from three additional blowfly species, *L. sericata*, *Cochliomyia hominivorax* and *C. macellaria*. The blowfly adult male and female transcripts differ in the choice of splice donor site in the first intron, with males using a site downstream of the site used in females. The *tra* genes all contain a single TRA/TRA2 site in the male exon and a cluster of four to five sites in the male intron. However, overall the sex-specific intron sequences are poorly conserved in closely related blowflies. The most conserved regions are around the exon/intron junctions, the 3′ end of the intron and near the cluster of TRA/TRA2 sites. We propose a model for sex specific regulation of *tra* splicing that incorporates the conserved features identified in this study. In *L. sericata* embryos, the male *tra* transcript was first detected at around the time of cellular blastoderm formation. RNAi experiments showed that *tra* is required for female development in *L. sericata* and *C. macellaria*. The isolation of the *tra* gene from the New World screwworm fly *C. hominivorax*, a major livestock pest, will facilitate the development of a “male-only” strain for genetic control programs.

## Introduction

A Y-linked male determining gene (*M*) determines sex in the Australian sheep blowfly *Lucilia cuprina* [1]. A likely target of *M* is the *transformer* (*tra*) gene, which is required for female development in *L. cuprina* [2]. The *L. cuprina tra* gene is sex-specifically spliced such that only the female transcript encodes a full-length functional protein. A very similar mechanism determines sex in the housefly [3] and tephritid fruit flies [4]–[6]. In *Drosophila melanogaster*, *tra* is required for female development but *tra* RNA splicing is regulated by the female-specific RNA binding protein SXL [7]. In the honeybee *Apis mellifera*, the apparent *tra* ortholog *feminizer* (*fem*) is regulated by heterozygosity of the *complementary sex determiner gene* (*csd*) [8], [9]. *csd* heterozygotes are females, whereas homozygotes and hemizygotes develop as males. *fem* transcripts are sex-specifically spliced with only the female transcript coding for FEM protein. *fem* RNA could be directly regulated by CSD, which is a putative splicing factor [10]. An ortholog of *tra* is required for female development in the hymenopteran *Nasonia vitripennis*, but *tra* gene expression appears to be controlled by a maternal imprinting mechanism rather than a male-determining gene [11].

In *L. cuprina*, the female and major male *tra* transcripts differ only in the choice of splice donor site for the first intron. The female site is 311 bp upstream of the male splice donor site. The male exon contains multiple in-frame translation stop codons. The TRA protein encoded by the female transcript contains a high proportion of serine and arginine amino acids, characteristic of SR splicing regulators [12]. Indeed, the *D. melanogaster* TRA protein is a potent enhancer of RNA splicing [13]. In *D. melanogaster,* TRA together with the RNA binding proteins TRA2 and RBP1, regulates the RNA splicing of transcripts from the *doublesex* (*dsx*) and *fruitless* (*fru*) genes [14]. With regard to *dsx*, the TRA/TRA2/RBP1 complex binds to six very similar 13 nucleotide sites within the 3′UTR of the female transcript and enhance the use of a nearby weak splice acceptor site [15], [16]. The binding sites are often referred to as TRA/TRA2 sites or the *dsx*RE. TRA appears to regulate *dsx* RNA splicing in *L. cuprina* in a similar manner as eight predicted TRA/TRA2 sites were found in the female-specific exon [17]. In support of this model, the RNA recognition motif (RRM) of the *L. cuprina* TRA2 ortholog is very similar to the RRMs of the *Drosophila*, tephritid and housefly TRA2 proteins [2]. The presence of a cluster of four predicted TRA/TRA2 sites within the first intron and another site in the male exon of the *L. cuprina tra* gene suggested that *tra* RNA splicing is likely autoregulated [2]. *tra* RNA splicing also appears to be autoregulated in the housefly [3] and tephritid fruit flies [4], [5], [18]. The presence of maternally inherited *tra* RNA transcripts could provide sufficient TRA protein to initiate the autoregulation of zygotic *tra* RNA splicing. This model of maternal activation of *tra* was first proposed in *C. capitata* [4] and recently confirmed for *M. domestica tra* [3].

The expression of *M* in male embryos somehow inhibits the female mode of *tra* RNA splicing. It is not known how *M* regulates *tra* splicing in *L. cuprina*, housefly or tephritid fruit flies but several possible mechanisms have been proposed [2]–[4]. *M* could encode a protein that directly interacts with TRA and inhibits its function. In support of this model, the blowfly, housefly and tephritid fruit fly TRA proteins contain a highly conserved amino terminal domain that is absent from the Drosophila TRA protein [2]–[4]. Alternatively, M could bind to *tra* RNA transcripts and either inhibit the female-specific splice or enhance the male-specific splice. For example, M could inhibit the binding of TRA/TRA2 to *tra* transcripts. If *M* interacts with the *tra* RNA transcript, then it would be predicted that sequence motifs in addition to the TRA/TRA2 sites would be conserved in the *tra* genes of related species. With the aim of identifying motifs that may play a role in *tra* sex-specific splicing, in this study we have isolated and compared the *tra* gene sequences from three blowfly species, *L. sericata*, *Cochliomyia hominivorax* (New World screwworm) and *C. macellaria* with the *tra* gene from *L. cuprina*. Based on molecular phylogenetic analyses, *L. sericata* is the closest relative of *L. cuprina* in the subfamily Lucilinae [19], [20]. Similarly, *C. hominivorax* and *C. macellaria* are very closely related but belong to the Chrysomyinae subfamily. We find that the blowfly *tra* genes have a similar organization and contain conserved motifs that may be important for sex-specific splicing.

## Results

### Organization and Conservation of the *C. hominivorax*, *C. macellaria* and *L. sericata tra* Genes

**Figure 1:**
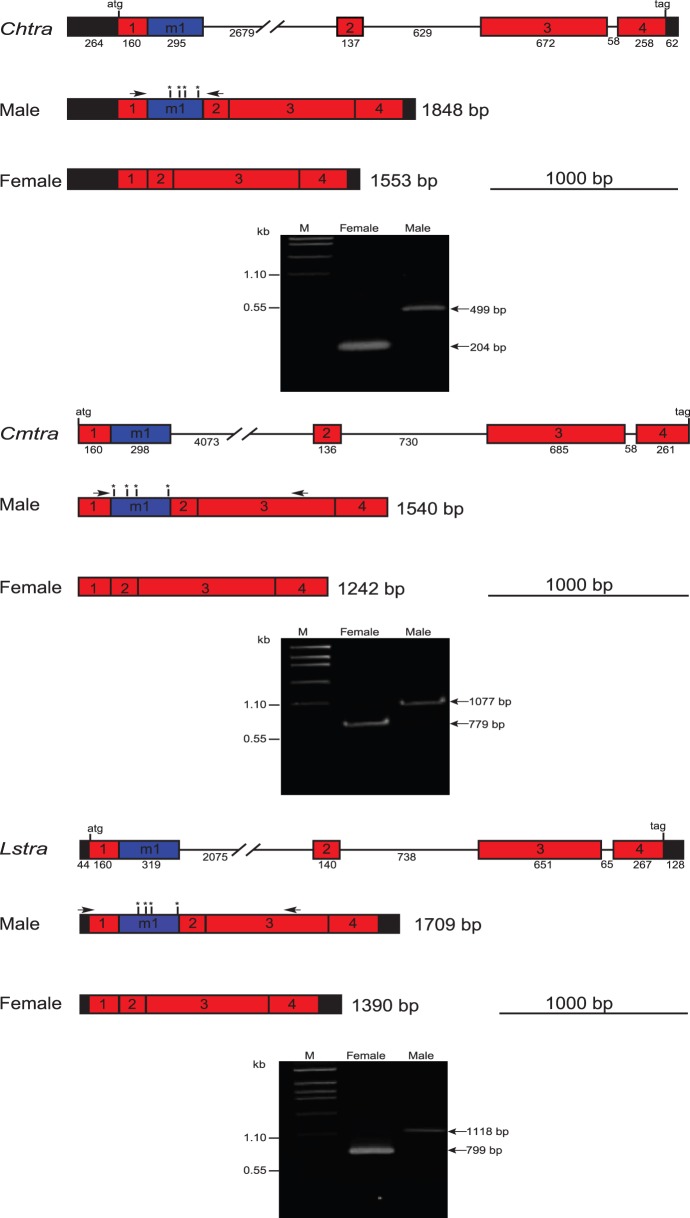
*tra* gene organization and sex-specific transcripts in *C. hominivorax* (*Ch*), *C. macellaria* (*Cm*) and *L. sericata* (*Ls*). *tra* genes of all 3 species consist of 4 exons (red boxes) and one male specific exon (blue box). Introns are shown with a black line. All exons and introns are drawn to scale with intron 1 interrupted as it significantly larger than the other introns. 5′ and 3′ untranslated regions are shown in black boxes. Exon and intron sizes in bp are shown. Male and female transcripts are represented below the gene. Vertical lines with asterisks indicate the relative locations of in-frame stop codons in the male exons. Arrows indicate the primer pairs used for RT-PCR. A single predominant product was obtained from each sex (arrows).

To isolate the *tra* gene from *C. hominivorax* and *C. macellaria* (*Chtra* and *Cmtra*), we used the same strategy as used previously to isolate the *L. cuprina tra* gene (*Lctra*) [2]. RT-PCR was performed using degenerate primers based on conserved amino acid motifs in the TRA protein to obtain a fragment of the *tra* transcript. For *L. sericata*, *tra* contigs had been identified in several transcriptome assemblies [21]. Additional RT-PCR reactions and 5′ and 3′ RACE were performed with gene specific primers and male and female RNA templates to obtain full-length (*Chtra* and *Lstra*) or near full-length (*Cmtra*) transcript sequences. We found only one major transcript in males and one in females in all three species (Figure 1). The male transcripts have an additional exon with several in-frame stop codons. The genomic organization of the *tra* genes was determined by comparison of cDNA and genomic DNA sequences. All the *tra* genes have an identical exon/intron organization, with four protein-coding exons, three introns and one male-specific exon (Figure 1). The TRA proteins encoded by the female-specific transcripts are highly similar (Figure S1). Previously identified domains (e.g. arginine/serine rich) are particularly well conserved. In pairwise comparisons, the TRA proteins from species within the same genus were 91 to 92% identical and 92 to 94% similar (identical plus conservative amino acids). However, the TRA proteins were only 67 to 70% identical and 78 to 80% similar when comparing *Cochliomyia* with *Lucilia* TRA proteins. Similarly at the nucleotide level, the *tra* coding regions are 91 to 93% identical within the same genus but only 73–74% identical outside the genus.

**Figure 2:**
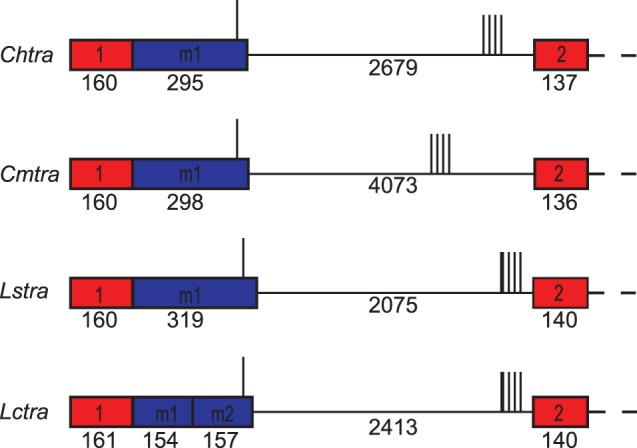
Comparison of blowfly *tra* alternatively spliced intron one and flanking exons. Exon 1 and exon 2 are shown in red boxes. One male specific exon in *Chtra*, *Cmtra*, *Lstra* and two male exons in *Lctra* are shown in blue boxes. Vertical lines indicate the locations of putative TRA/TRA2 binding sites.

The male and female transcripts arise from alternative splicing, differing in which splice donor site is used to join with the intron 1 splice acceptor site (Figure 2). We previously suggested that *tra* alternative splicing in *L. cuprina* is likely to be autoregulated as 5 motifs that matched a TRA/TRA2 consensus sequence (U/AC/AA/UA/UCAAUCAACA) were identified in the intron and one TRA/TRA2 site was in male exon M2. The five sites in the intron were clustered towards the 3′ end. *tra* splicing is likely auto-regulated in *C. hominivorax*, *C. macellaria* and *L. sericata* as in all three species there are either four (*Chtra*, *Cmtra*) or five (*Lstra*) TRA/TRA2 sites within the regulated female first intron. Moreover, the relative location of the TRA/TRA2 sites is very similar to that in *Lctra*, with one TRA/TRA2 site towards the 3′ end of the male exon and a cluster of sites in the intron. In *Lstra* and *Chtra*, the TRA/TRA2 cluster is towards the 3′ end of the intron as in *Lctra*, whereas in the larger *Cmtra* intron the sites are more towards the middle of the intron.

In contrast to the well-conserved TRA/TRA2 sites, the male first introns and male exons show little overall nucleotide sequence similarity. The sequence identity of the male intron ranged from 56% (*Lctra*/*Lstra*) to 28% (*Cmtra*/*Lstra*), the low identity of the latter is partly because of an insertion in the *Cmtra* intron (see below). The male exon was more conserved with highest similarity within a genus (85% *Cochliomyia*, 79% *Lucilia*), than between genera (47 to 50%). To identify motifs that may play a role in the regulated splicing of the first intron, we searched for conserved sequences by performing pairwise comparisons, multiple sequence alignments and by MEME analyses.

**Figure 3:**
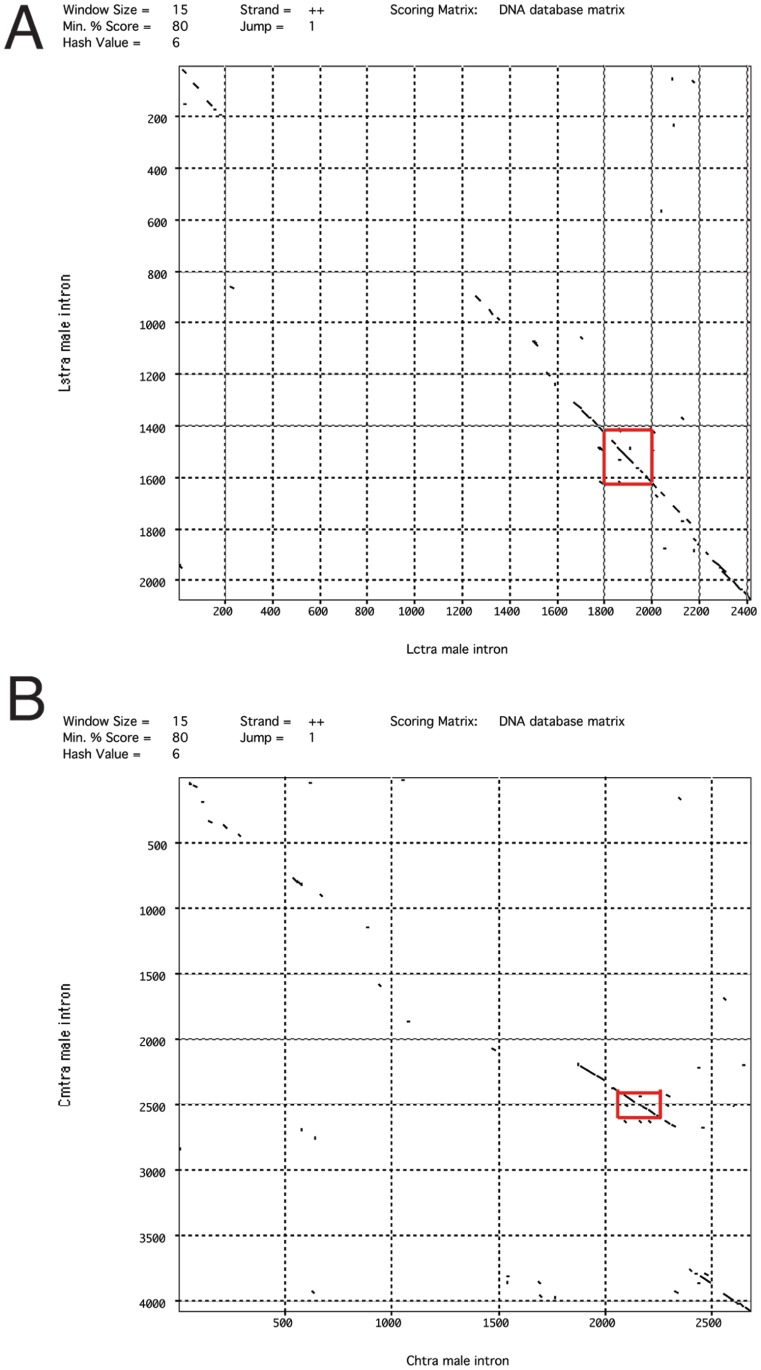
DNA dot matrices of blowfly *tra* male intron DNA sequences. (A). A comparison of *Lucilia tra* intron sequences with *Lstra* on the vertical axis and *Lctra* on the horizontal. (B) A comparison of *Cochliomyia tra* intron sequences with *Cmtra* on the vertical axis and *Chtra* on the horizontal. The regions containing the cluster of TRA/TRA2 sites are highlighted with a red box.

**Figure 4:**
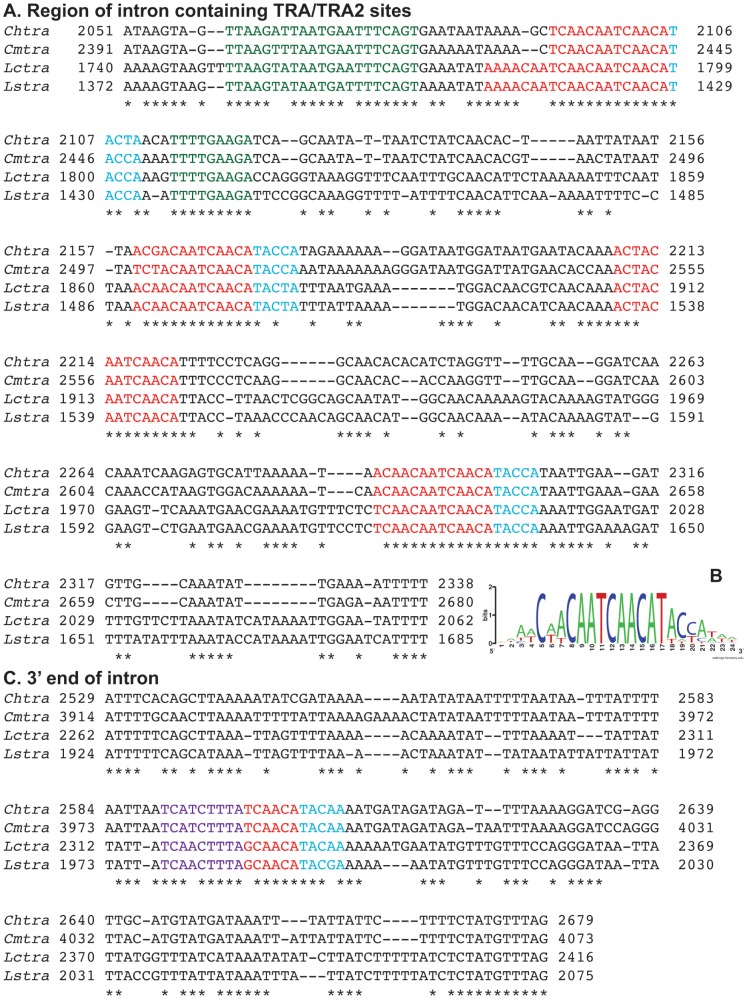
Multiple sequence alignments of *tra* male introns. (A) Region containing the conserved TRA/TRA2 sites, which are shaded red. The TACC/TA motif is shaded blue after the TRA/TRA2 sites and the conserved motifs near the most 5′ TRA/TRA2 site are shaded green. (B) Graphical representation of a multiple sequence alignment of all of the TRA/TRA2 sites from the *Chtra*, *Cmtra*, *Lctra*, *Lstra* and *Lcdsx* genes. (C) Alignment of the sequences at the 3′ end of the intron. A conserved RBP1 type A binding site is highlighted in purple, the 3′ half of a TRA/TRA2 site in red and the TACC/TA motif in blue.

Pustell DNA matrix analyses of the male intron DNA sequences were undertaken to obtain a visual representation of how the sequences were related. Only the comparisons within a genus showed significant blocks of similarity. The conserved blocks were clustered at the ends of the introns, particularly the 3′ end and also around and including the cluster of TRA/TRA2 sites (Figure 3). This analysis also revealed an insertion of about 1100 bp towards the 3′ end of the *Cmtra* intron, which has separated the TRA/TRA2 cluster from the splice acceptor site. We then performed multiple sequence alignments, focusing on the region of the intron containing the TRA/TRA2 sites and also the 3′ end of the intron. The spacing between the TRA/TRA2 sites is generally well conserved with only a few minor insertions/deletions (Figure 4A, red). In the *Lucilia* species, the most 5′ site contains two overlapping motifs that match the TRA/TRA2 consensus. Three of the four TRA/TRA2 sites are followed by the sequence TACC/TA (Figure 4A, blue). This sequence is also found after the single TRA/TRA2 site in the male exon (Figure S2). This suggests that the consensus TRA/TRA2 site in blowflies may be longer than the 13 bp motif identified previously [2]. If so, the eight TRA/TRA2 sites in the 3′ UTR of the *L. cuprina* female *dsx* transcript [17] should be followed by the TACC/TA motif. Indeed, the motif is immediately 3′ of four of the eight sites. Furthermore, all eight sites are followed by a TAC sequence. A logo derived from an alignment of 26 bp sequences containing the predicted TRA/TRA2 sites in the blowfly *tra* genes and the *Lcdsx* gene is shown in Figure 4B. An optimal motif of 18 to 19 bp is clearly seen from inspection of this graphical representation of the alignment. In addition to the TRA/TRA2 sites, there are two motifs near the most 5′ TRA/TRA2 site that are well conserved (Figure 4A, green). Neither of the motifs contains a predicted binding site for RBP1 or the TRA2-ISS site, previously suggested to be important for *tra* sex-specific splicing in tephritids [18]. We next asked if the motifs contain recognition sites for any other *Drosophila* RNA binding protein for which the RNA binding protein specificities are known. The only hit was to a binding site for the dUNR protein within the 3′ UTR of the *msl-2* transcript [22]. The sequence, AUGAAUUU, is within the motif upstream of the most 5′ TRA/TRA2 site. However, dUNR binding to *msl-2* RNA requires the binding of SXL to an adjacent polyU sequence [22], which is not present in the *tra* intron. Thus, it would appear unlikely that UNR would bind to the conserved motif in the *tra* intron. Nevertheless, the possibility that UNR binds to *tra* transcripts should not be completely discounted given that UNR binds to many sex-specifically processed transcripts in *Drosophila* [23].Interestingly, the motif downstream of the most 5′ TRA/TRA2 site shows a partial (7/8 in *Chtra*) match to the sequence [(5′-(U/G)GAAGAU(U/A)-3′] suggested by Verhulst et al [11] to be a potential binding site for TRA/TRA2 in *Nasonia vitripennis* and *Apis mellifera*. Alignment of the DNA sequences of the 3′ end of the intron identified a conserved type A RBP1 binding site (DCADCTTA) about 100 bp upstream of the splice acceptor site (Figure 4C, purple). This was immediately followed by a conserved motif that matched the 3′ half of the extended TRA/TRA2 site (red). A multiple sequence alignment of the male exon sequences identified the conserved TRA/TRA2 site near the 3′ end but there were few other blocks of high conservation apart from the splice donor site (Figure S2). A short T-rich motif occurred about 40 bp downstream of the splice donor site (green). The binding site for the neuronal protein ELAV in the *Drosophila ewg* RNA is characterized by short and variably spaced polyU motifs [24]. Additional short T-rich motifs occur elsewhere in the male exon including the 5′ end (*i.e.* female splice donor site) and 3′ to the TRA/TRA2 site. ELAV regulates the alternative splicing of *ewg* transcripts in neurons. Perhaps the short U-rich motifs are part of the binding site for a constitutively expressed ELAV-like protein in calliphorids.

**Figure 5:**
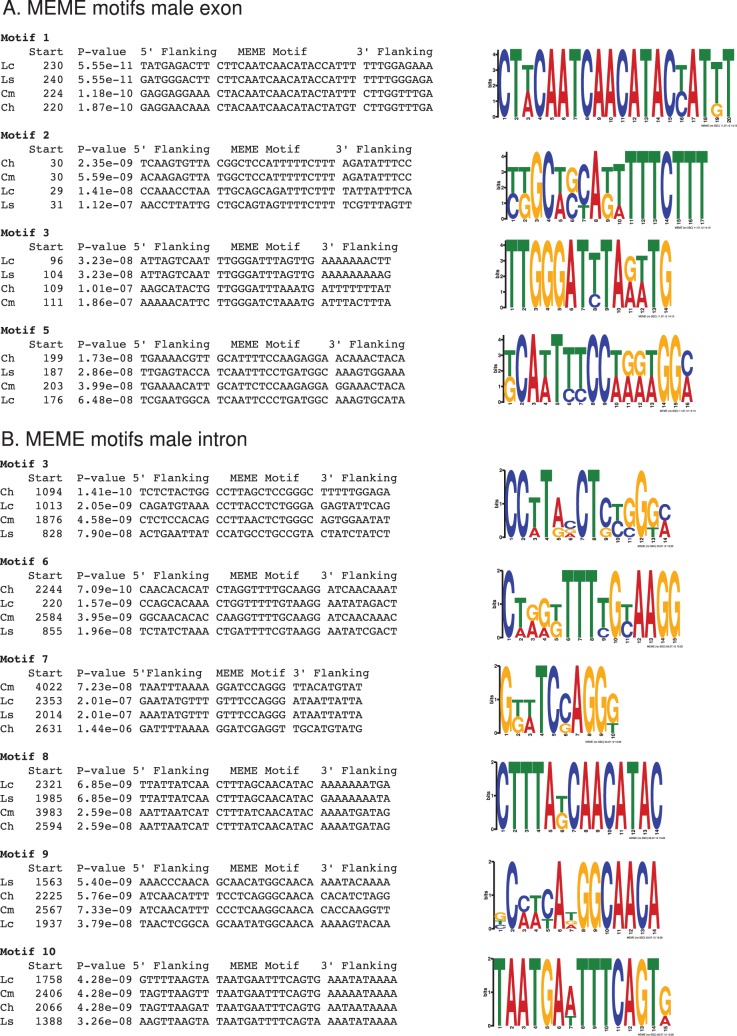
Conserved motifs in the male exons and introns identified by MEME analyses. (A) Motifs in male exon and (B) motifs in the male intron. The start of the 3′ flanking sequences of the motifs is shown relative to the start of either the exon (A) or intron (B). *Ch, C. hominivorax; Cm, C. macellaria; Ls, L. sericata; Lc, L. cuprina.*

We used MEME to search for sequence motifs in the male exons and male introns that are conserved at the nucleotide level but not necessarily occur at similar positions (Tables S1 and S2). In the male exon, the highest ranked motif contained the single TRA/TRA2 site (Figure 5A). Motifs 2 and 3 were located about 30 and 100 bp downstream from the start of the male exon respectively and did not contain RBP1 or ISS sites. Motif 2 contained the T-rich sequence identified above whereas Motif 4 contained the female splice donor site and thus was of less interest (Table S1). Motif 5 was located about 30 bp upstream from the single TRA/TRA2 site (Figure 5A). None of the remaining motifs had low *p*-values in all four species. Of the ten highest ranked motifs identified in the male introns, four of the top five contained a TRA/TRA2 site (Table S2). Motif 3 has a relatively high GC content and was located in a region of the intron that is otherwise poorly conserved (Figure 5B). Motif 6 is T-rich, occurs at various locations within the intron and the 3′ end matches the *Drosophila* ISS site. Motifs 7 and 8 are found near the 3′ end of the intron. Motif 8 contains part of the RBP1 type A identified by sequence alignments whereas motif 7 is located between this motif and the splice acceptor site. Motifs 9 and 10 occur within the cluster of TRA/TRA2 sites with 9 downstream of the third site and 10 upstream of the first TRA/TRA2 site. Motif 10 contains the conserved sequence previously identified (Figure 4A). Neither the motifs identified by MEME analysis or multiple sequence alignment contained binding sites for other *Drosophila* RNA binding proteins for which the RNA binding protein specificities are known (http://rbpdb.ccbr.utoronto.ca/).

We next asked if any of the conserved motifs occur in *tra* genes in non-calliphorids. We searched the tsetse fly (*Gmmtra*) and housefly (*Mdtra*) *tra* genes as these are the closest relatives for which complete gene sequences are available. About 35 bp upstream of the cluster of TRA/TRA2 sites in the *Gmmtra* first intron is a sequence (5′-AATTTCTGTAGAA-3′) that is a 10/13 match to the conserved sequence that is upstream of the first TRA/TRA2 site in the male intron of calliphorid *tra* genes (Figure 4A). Of the motifs identified by MEME analysis, only motif 5 in the male exon (Figure 5A) was found in the *Gmmtra* gene. The sequence (AAAATATATTGGATGT) occurs within the sex-specific female intron, but not within any of the male exons [3].

**Figure 6:**
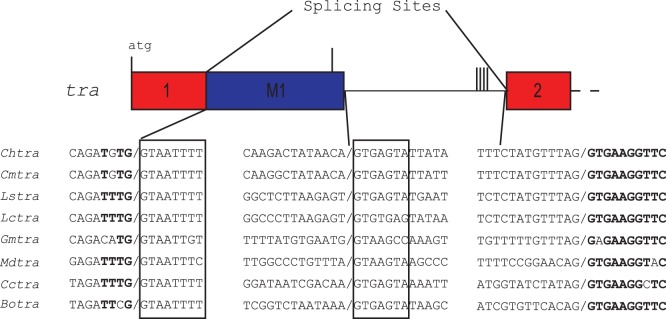
Comparison of tra sex-specific intron splice donor and acceptor sites from eight fly species. C.h: C. hominivorax. C.m: C. macellaria. L.s: L. sericata. L.c: L. cuprina. G.m: G. morsitans. M.d: M. domestica (house fly). C.c: C.capitata and B.o.: B. oleae. The highly conserved female and male splice donor sites are boxed, conserved exon sequences are in bold.

There is considerable conservation around the splice donor and acceptor sites of the alternatively spliced first intron (Figure 6). The female donor site sequence TG/GTAATTTT is invariant in the four blowfly species. The sequence is also highly conserved in tsetse fly, housefly and tephritid fruit flies. In all of these species a male-specific exon immediately follows the first protein coding exon. The G at the -1 position is the first nucleotide of a codon for a highly conserved glycine (first Gly in sequence discussed below). The T at the -2 position is in the third position of a cysteine (ChTRA, CmTRA) or phenylalanine (LsTRA, LCTRA) codon and so would not be expected to be so highly conserved. The male donor site is also highly conserved and in all species the sequence shows a strong match to the *Drosophila* consensus sequence (AG/GTAAGT) [25]. Indeed, when we analyzed the *tra* gene nucleotide sequences using a *Drosophila* splice site prediction program (NNsplice) [26], the male splice site donor site received a stronger prediction score. For example, for *Chtra*, the scores were 0.93 and 0.47 for the male and female donor sites respectively (a score of 0.4 is the cutoff for significance). The splice acceptor sites also all generally match the Drosophila consensus. The exon sequence that follows the acceptor site, GTGAAGGTTC is invariant in blowflies and highly conserved in the other fly species we examined (Figure 6). This may be partly because the amino acids encoded by the exon sequence (Gly-Glu-Gly-Ser) are highly conserved. Nevertheless, some nucleotide variation might have been expected at the variable third position of the codons. This conservation suggests that the conserved exonic sequences adjacent to the female splice donor and acceptor sites may be important for efficient sex-specific splicing. In *L. cuprina*, we previously found one major and one minor male transcript. The major transcript *Lctra* transcript corresponds to the male transcript identified in the other blowfly species. The minor *Lctra* transcript arises through an additional splicing event that excises exon M1 (Figure 2). The splice acceptor site in exon M1 is not well conserved in *L. sericata*, differing at 8 of 14 positions (not shown), which may be why the minor splice variant was not detected in *L. sericata*.

### *tra* Transcripts are Sex-specifically Spliced Early in Development in *L. sericata*

**Figure 7:**
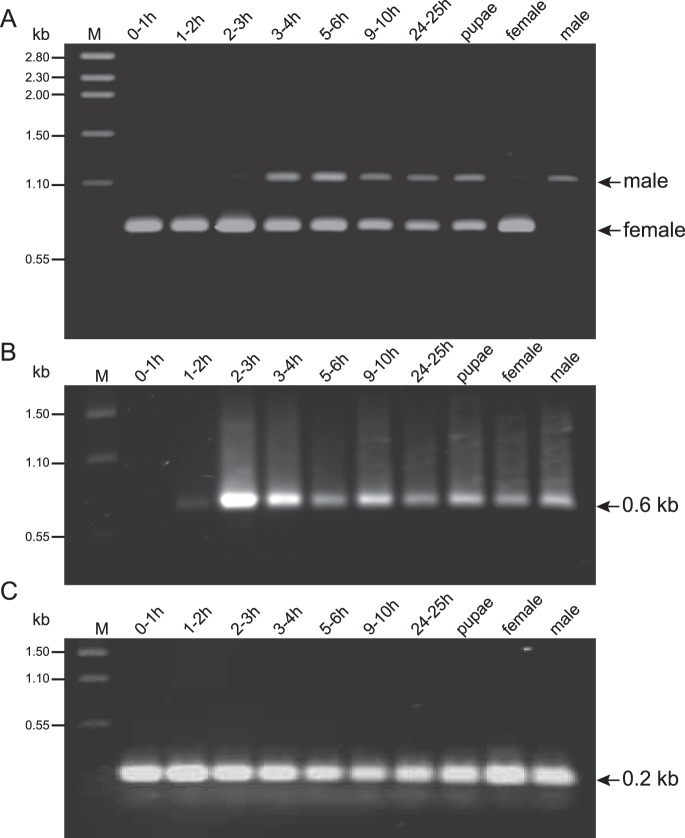
Developmental expression of *Lstra*. Total RNA was extracted from *L. sericata* mixed sex embryos and pupae and adult male and female. The embryos were collected within one hour of laying then aged for the indicated period at room temperature. (A) RT-PCR with *Lstra* primers yielded a 1.1 kb male product and 0.8 kb female product as indicated. The position of the primer pair used for RT-PCR is shown in Figure 1. (B) RT-PCR product of the cellularization gene *Lsslam* and (C) *Lsmsl2* as a loading control.

In *L. cuprina* [2], housefly [3] and medfly [4], both male and female precellular embryos initially only contain the female form of *tra* RNA inherited from their mother. In housefly and medfly, the male form of *tra* RNA first appears in precellular embryos after the initial onset of zygotic gene expression [3], [27]. In *L. cuprina*, we were unable to detect either male form of *tra* RNA in embryos [2]. If the male transcript was present at a significantly lower level than the female transcript this could have made detection difficult. Additionally, we may have been unable to detect the male product if the shorter female product was preferentially amplified with mixed-sex RNA template. We therefore repeated the developmental expression profile with *L. sericata* with the rationale that because the nucleotide sequence of the male exon is significantly different (79% identity to *L. cuprina*), any amplification bias (if it exists) may be reduced. RT-PCR was performed from carefully staged mixed-sex embryos and also pupae and adults. Male product was readily detected in embryos collected 3 to 4 hours after egg laying and in all later staged embryos (Figure 7A). In addition, relatively low amounts of male product were present in RNA from 2–3 h old embryos. Although the embryos were presumably an equal mix of males and females, there was stronger amplification of the shorter female product. This suggests that the male transcript is present at a lower level and/or there is preferential synthesis of the female product either because of an amplification bias or more efficient reverse transcription. Cellular blastoderm formation was observed around 2 h of development. Consistent with this observation, expression of the cellularization gene *Lsslam* was first detected in 1–2 h embryos and readily seen in 2–3 h embryos (Figure 7B) Thus in *L. sericata*, the zygotic male-specific *tra* splicing occurs early in development around the time of cellularization.

### *tra* is Essential for Female Development in *L. sericata* and *C. macellaria*

**Figure 8:**
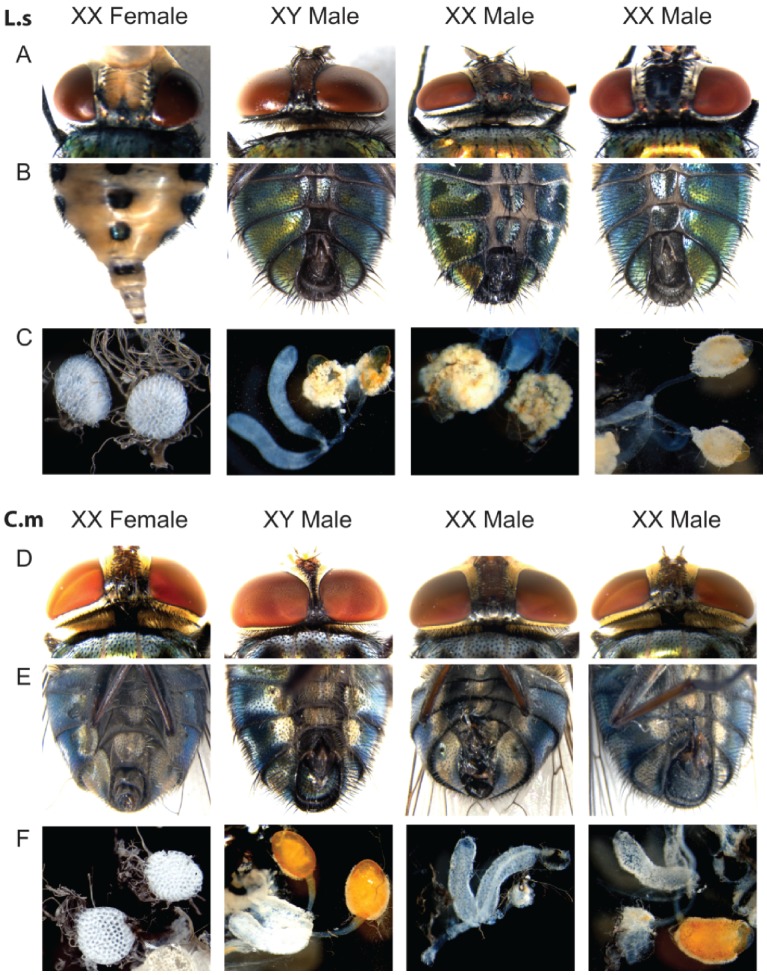
*tra* RNAi mediated partial sexual transformation of *L. sericata* and *C. macellaria.* Head (A, D) and genitalia (B, E) of wild type XX female, XY male and XX partially transformed males in *L.sericata* (A–B) and *C. macellaria* (D–E). (C, F) Dissection of adult XX female, XY male and XX partially transformed males in *L.sericata* (C) and *C. macellaria* (F). The transformed XX males develop testes-like structures.

Injection of a 950 bp double-stranded *Lctra* RNA into the posterior end of preblastoderm *L. cuprina* caused about three quarters of the XX individuals to develop some male features [2]. Of the XX flies, 2% showed complete sex reversal to male whereas most (72%) developed with external male genitalia but with a wide interocular width characteristic of females. In the absence of any molecular markers to distinguish XY males from transformed XX males, we used the partial sex transformation phenotype to determine if *tra* is required for female development in *L. sericata* and *C. macellaria.* About 300 preblastoderm *L. sericata* and *C. macellaria* embryos were injected with a 799 bp *Lstra* or 851 bp *Cmtra* doublestranded RNA respectively. Partially transformed XX individuals were identified based on the appearance of male genitalia but female interocular width (Figure 8A, B and D, E). From the *L. sericata* embryos injections, we obtained 42 females with normal genitalia and 10 partially transformed XX males. Similarly, 18 females and 8 partially transformed XX males developed from the *C. macellaria* embryos injected with *Cmtra* dsRNA. The partially transformed XX males showed evidence of development of testes and not ovaries (Figure 8C, F). We conclude that *tra* is required for female development in *L. sericata* and *C. macellaria.*

## Discussion

The *transformer* genes from the blowfly species *L. cuprina*, *L. sericata*, *C. hominivorax* and *C. macellaria* have a remarkably similar arrangement of exons/introns and TRA/TRA2 sites. In all species the major male and female transcripts arise from a different choice of donor site for the splicing out of the first intron. In contrast, the *transformer* genes from the tephritid fruit flies *C. capitata*, *B. oleae* and *A. obliqua* have complex splicing patterns, several male-specific exons and differ in the number and relative location of TRA/TRA2 sites [4], [5], [18]. The blowfly *tra* genes all contain a single TRA/TRA2 site near the 3′ end of the male exon. Interestingly, there is also at least one TRA/TRA2 site in the male exon that is immediately downstream of the first protein coding exon (exon 1 in blowfly *tra* genes) in the tephritid, housefly and tsetse fly (*G. morsitans morsitans*) *tra* genes [3]–[5], [18]. This suggests that binding of TRA/TRA2 to that male exon likely plays an important role in splice site selection in females in these species.

We found that the TRA proteins from within the same genus were highly similar (92–94%) but less similar when comparing outside the genus (78–80%). These results are consistent with previous morphological and phylogenetic studies that showed that the two *Lucilia* species are very closely related, as are the two *Cochliomyia* species [19], [20]. Similar results were obtained previously comparing TRA proteins from different tephritid species [18]. TRA proteins from within the genus *Anastrepha* were highly similar (88–99%), but TRA proteins from different genera were less similar (54–64%). In contrast to the high similarity of the coding regions, overall the nucleotide sequences of the regulated first intron of the calliphorid *tra* genes were rather poorly conserved. However, this highlighted several conserved sequence blocks that are likely important for sex-specific splicing. The nucleotide sequences showed particularly high similarity around the cluster of four TRA/TRA2 sites, the exon/intron junctions and the 3′ end of the intron. The nucleotide sequences adjacent to the first TRA/TRA2 site (i.e. most 5′) were particularly well conserved in blowflies. These sequence motifs could potentially be binding sites for other proteins that could interact with TRA or TRA2. Our analysis of the TRA/TRA2 binding sites led to the realization that the optimal binding site may be up to 5 nucleotides longer than the 13 nt motif originally identified. In *Drosophila*, RBP1 binds to the 5′ half and TRA2 to the 3′ half of the 13 nt repeat (i.e. TRA/TRA2 site) in the *dsx*RE [16]. We identified a conserved RBP1 type A binding site near the 3′ end of the first intron, which was followed by the 3′ half of a longer TRA/TRA2 site. This suggests that this conserved motif could be bound by RBP1 and TRA2 and may play an important role in regulated splicing of the *tra* first intron. MEME analyses identified several other motifs in the male exons and male introns that could play a role in *tra* splicing. Lastly, we observed that the nucleotide sequences around the female and male splice donor sites were highly conserved in all dipteran species where *tra* splicing is autoregulated. Such conservation suggests there is strong selection pressure to maintain the sex-specific donor site nucleotide sequences. In calliphorids, the male splice donor site received a higher score with splice site prediction software, suggesting this site would be preferred by the spliceosome.

**Figure 9:**
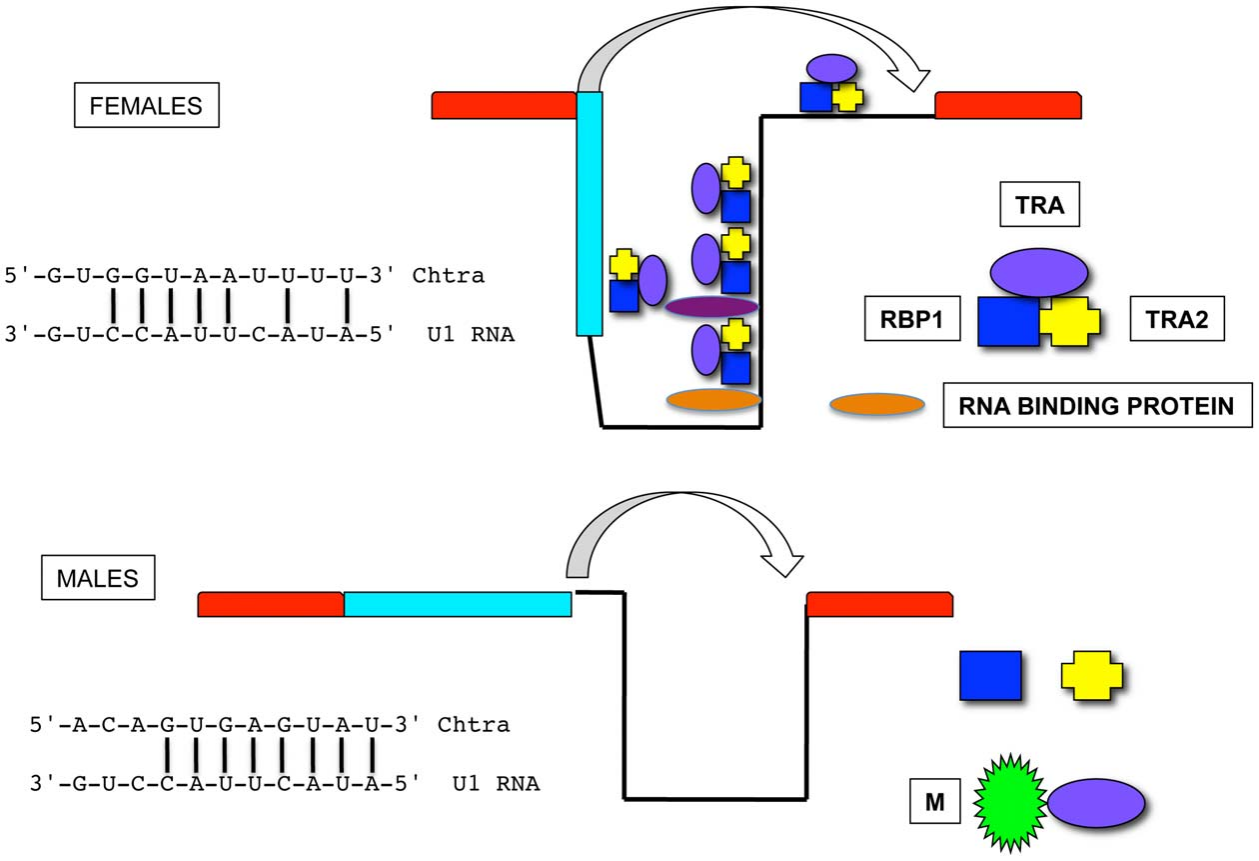
Proposed model for regulation of *tra* splicing in calliphorids. The male splice donor site is preferred in males, possibly because this forms a stronger predicted hybrid with U1 snRNA (*Drosophila* sequence shown) than the female donor site. M is suggested to bind to the conserved amino terminal domain of TRA and prevent assembly of the TRA/TRA2/RBP1 complex. See discussion for alternative mechanisms by which M could regulate *tra* splicing. In females the TRA/TRA2/RBP1 complex binds to the single site in the male exon and the cluster of sites in the intron. We propose that intermolecular interactions between bound complexes block use of the male splice donor site. This interaction may be facilitated by additional RNA binding proteins that bind to conserved motifs (Figures 4,5). A TRA/TRA2/RBP1 complex is suggested to bind to the conserved motif (Figure 4B) near the 3′ splice acceptor site and enhance splicing with the female splice donor site.

We propose a model for sex-specific regulation of *tra* splicing in calliphorids that takes into account the conserved features identified in this study (Figure 9). We suggest that in females, TRA/TRA2/RBP1 bound to the site in the male exon and the sites in the intron associate and prevent access of the spliceosome to the male splice donor site. Association of TRA/TRA2/RBP1 complexes could be mediated by the proposed auto regulation domain that occurs near the amino end of TRA in non-drosophilid Diptera [11]. Other RNA binding proteins bound to conserved motifs near the most 5′ TRA/TRA2 site in the intron and elsewhere in the intron may contribute to the inhibition of the male splice donor site. In addition TRA/TRA2/RBP1 bound to the site near the splice acceptor site could enhance splicing to the female donor site. In males, M interferes with TRA/TRA2/RBP1 regulation of splicing. M could bind to one or more of the conserved motifs in the intron and directly interfere with RNA binding and/or association of TRA/TRA2/RBP1 complexes. Another possibility is that M represses *tra* gene expression, which would be supported by the relatively low levels of the male form of *tra* transcript we detected in *L. sericata* embryos. Alternatively, M may bind to the auto regulation domain and directly interfere with TRA/TRA2/RBP1 complex assembly (Figure 9). The latter is consistent with preliminary data that the *Chtra* intron is sex-specifically spliced in *D. melanogaster* (results not shown), which does not use M to determine sex. In our model, the male splice site is utilized as this is preferred by the splicing machinery. This may be because the splice donor site can form an 8 bp double stranded hybrid with *Drosophila* U1 RNA (Figure 9) [28]. The female splice donor site forms a less perfect match with the U1 RNA. Although this model is clearly highly speculative it does make several predictions that could be tested using functional assays. For example:

1. Exchange of the male and female splice donor sites would result in both sexes using the most 5′ splice donor site (i.e. the female site in the wild type *tra* gene).
2. The male splice donor site would be preferred over the female donor site in splicing assays with extracts from Drosophila S2 cells (male) [13].
3. The TRA/TRA2 site in the male exon would be particularly important for splicing regulation
4. The TRA/TRA2/RBP1 complex would bind to the site near the splice acceptor site and this binding would be important for splicing efficiency in female calliphorids.

Similar to *Drosophila*, *Lucilia* embryos develop rapidly reaching the cellular blastoderm stage around 2 h after egg laying. In contrast, in *C. capitata*, cellular blastoderm formation occurs between 9 and 11 h after oviposition [27]. Taking advantage of this elongated precellularization phase, Gasperi and colleagues found that between 4 and 8 h of development both male and female embryos express several *Cctra* transcripts, including the adult male and female forms [27]. By 9 h of development male and female embryos express only the sex-specific *Cctra* transcripts. They proposed that M acts during the critical precellularization phase when maternal CcTRA protein is not sufficient to direct only the female mode of splicing. Because *Lucilia* embryos develop rapidly it is difficult to say if male transcripts appear prior to cellular blastoderm formation. Male *Lstra* transcripts were first detected, albeit at relatively low levels, in 2–3 h embryos. Transcripts from the cellularization gene *Lsslam* were first detected at 1–2 h and were readily apparent by 2–3 h. Thus M appears to act early in *Lucilia* embryos around the time of cellular blastoderm formation. Consistent with an early setting of *tra* sex-specific expression in blowflies, we found that injection of *tra* dsRNA into the posterior end of *L. sericata* and *C. macellaria* embryos led to partially masculinized adults that had male genitalia but female interocular width. These experiments confirmed the importance of TRA in female development in *Lucilia* and *Cochliomyia* species.

*C. hominivorax*, the New World screwworm fly, is a devastating pest of livestock [29]. The sterile insect technique (SIT) was used to eradicate *C. hominivorax* from North and Central America [30], [31]. This involved the mass rearing and subsequent release of sterilized male and female flies over the targeted area. SIT has also been successfully used to control populations of medfly [32]. The development of medfly genetic sexing strains enabled programs to release only sterilized males [33]. Such single sex releases are more effective for population reduction than bisexual releases [34], [35]. The isolation of the *Chtra* gene will facilitate the development of transgenic strains that carry repressible female lethal genes. For example, the female first intron (which includes the male exon) could be inserted into a lethal gene such that only the female transcript codes for a functional protein [36]. Expression of the sex-specific lethal gene would be under the control of the tetracycline dependent transactivator or tTA, which can be simply repressed by adding tetracycline to the diet [37]. By inserting the sex-specific intron from *C. capitata tra* (*Cctra*) within the tTA gene, Alphey and colleagues developed an auto-regulated single component tetracycline repressible female lethal system [36]. More recently, a two-component female embryo lethal system was developed in *A. suspensa* using the *Cctra* intron to confer sex-specific expression of the *Alhid* cell death gene [38]. In general, it appears that in order to assemble a system that functions very efficiently it is better to use components (promoter, sex-specific splicing, cell death) from endogenous genes (Li and Scott, unpublished observations) [38]. Thus by isolating the *Chtra* female intron, we have obtained an essential component for building a “male-only” strain of *C. hominivorax*. In designing the lethal gene construct our analysis suggests that it could be important to consider the exon sequences that flank the *Chtra* intron and that it may not be necessary to include the entire intron.

## Materials and Methods

### Fly Strains and Rearing

Adult *L. sericata* (strain CA06) and *C. macellaria* (strain NC09) were maintained at 22**°**C in wire frame cages (22×20×20 cm) covered with orthopedic stockinette. They were provided water and sugar ad libitum. Adult *L. sericata* were fed a protein rich gelled food (100 g/L whole egg powder, 50 g/L instant non-fat milk powder and 25 g/L inactivated dry yeast in 1.5% agar) on the second day following their eclosion. This gelled protein-rich food was refreshed daily for 5 consecutive days, then removed for 24 hours and replaced with a single dish of gelled food with narrow slits cut into it to provide crevices for oviposition. Eggs are collected from the cage and placed in containers of the gelled protein-rich food for larval rearing. For subsequent egg collections, the gelled food was removed after 3 consecutive days in the cage. The rearing procedures were similar for *C. macellaria*, except that eggs were collected on ground meat, adults were fed a sugar and protein-rich gelled food (150 g/L sugar, 100 g/L whole egg powder and 50 g/L instant non-fat milk powder in 1.25% agar) and larvae were reared on a protein-rich gelled food (100 g/L whole egg powder and 50 g/L instant non-fat milk powder in 1.5% agar). *L. sericata* and *C. macellaria* larvae were kept at 27**°**C in a forced air incubator.

Frozen *C. hominivorax* (strain J06) embryos and adults were obtained from the USDA-ARS screwworm research unit in Pacora, Panama.

### PCR, RACE, DNA Cloning, Bioinformatics and Accession Numbers

Total RNA from two adult males and females of each blowfly species were extracted with Trizol reagent (Sigma), purified by ethanol precipitation and subsequently treated with Turbo DNase (Ambion). Polyadenylated mRNA was further purified with GenElut Direct mRNA Miniprep Kit (Sigma). cDNA was synthesized with SuperScrip III First-Strand Synthesis SuperMix (Invitrogen). PCR conditions: denaturation at 95°C for 3 min, followed by denaturation at 95°C for 30 sec, annealing at 58°C for 30 sec, extension at 72°C for 1 min for 35 cycles; final extension at 72°C for 4 min. For 5′ and 3′ RACE, we used the Smarter RACE cDNA Amplification Kit and Advantage 2 Polymerase Mix (Clontech) according to the manufacturer’s instructions. Primer sequences used for RT-PCR and 5′ and 3′ RACE of *tra* are:

Ch-Tra-F1 5′-ATACCAAGTGGTTCGGTGAAAAGAGGTC-3′.

Ch-Tra-F2 5′-GTTTATATGGTCGTTCATGCTCTCGTTCACC-3′.

Ch-Tra-R1 5′-GGTTTTAGTTTTACCGCTTGTATGGTGTTC-3′.

Ch-Tra-R3 5′-CTGCTAGAGCGACTGTAGTGTTTGTGACGTT-3′.

Ls-Tra-F1 5′-ACCACAACAACTGCATATCATCGGCAACAAC-3′.

Ls-Tra F2 5′- ATTATATGGACGTTCACGTTCTCGTACACC-3′.

Ls-Tra-R1 5′-CGACGACTTCTATAGTCTCTTCTTACGTTACGG-3′.

Primers used for RT-PCR of *Lsslam* and *Lsmsl2* were:

Ls-SLAM-F: 5′-AGTGTTTTCTCTCAGCTATCGGTCAAGGAT-3′.

Ls-SLAM-R: 5′-ATCATTGCTATTATTAACGGGGGTAATGTCA-3′.

Ls-MSL2-F: 5′-ATTGTGCCCATAATGTTTGTCGTTTGT-3′.

Ls-MSL2-R: 5′-GAGCTATTGTATTGTCCCACTTTGTGTCC-3′.

Genomic DNA from 3 adult flies was extracted according to standard methods as previously described [39]. 50 ng of genomic DNA was used as template to amplify the *tra* intron. Primers are:

For the *Chtra* and also *Cmtra* intron : Ch-Tra-F1 and Ch-Tra-R1.

For the *Lstra* intron: Ls-Tra-F3 5′-ATTTAAAATTCAACAATCCATACCC-3′.

Ls-Tra-R2 5′-TCTAAATTATTAGTATCACGAGCAT-3′.

PCR and RT-PCR products were purified and cloned into pCR 4-TOPO vector using Topo TA Cloning kit for sequencing. Nucleotide sequences analysis and protein multiple sequences alignment were performed using Vector NTi (Invitrogen), MacVector and the Clustal Omega program. For MEME analyses [40], the male exon and male intron sequences were separately run using the OOPS model while restricting the search to motifs between 10 and 15 bp in length. Custom Python scripts were used to search all of the resulting MEME motifs for the consensus sequences of the TRA/TRA2, RBP1 and ISS binding motifs from *D. melanogaster*, *C. capitata*, *B. oleae* and *A. obliqua* [18]. Custom Python scripts were used to determine if available PFMs and IUPAC binding motifs of RNA-binding proteins from RBPDB (http://rbpdb.ccbr.utoronto.ca/) could be found within our motifs. For each PFM, PFMs were converted into regular expressions, which were in turn searched for within the sequences used to create our MEME motifs using the Python regular expression module’s search function. For each hit, the probability of that RNA-binding protein’s motif variant occurring was calculated using that protein’s binding motif PFM. All probabilities were low (<1e-6) suggesting the regular expression were very liberal in declaring matches but that the actual probability of the hit found within our sequences being that of the protein’s binding motif very low. For each IUPAC motif, it was converted into a regular expression and then broken down into overlapping substrings either of 10mers (for comparison to the sequences used to make our motifs) or 8mers (for the other requested motifs). These 10mers or 8mers were then searched against the sequences used to create our MEME motifs using the Python regular expression module.

The accession numbers for the genes reported in this study are:

*L. sericata transformer* gene, JX315620.

*C. macellaria transformer* gene, JX315619.

*C. hominivorax transformer* gene, JX315618.

### RNAi

*tra* dsRNA of *L.sericata* and *C. macellaria* were synthesized with either T7, T3, or SP6 RNA polymerase using a linearized plasmid DNA as a template as previously described [2]. For *L.sericata,* the plasmid DNA template contained a 799 bp female cDNA fragment including exon 1, 2 and 3. For *C. macellaria,* plasmid DNA was generated by cloning of a 851 bp of female cDNA fragment including exon 1, 2 and 3. in vitro synthesized RNA products were purified with phenol/chloroform extraction, ethanol precipitated and resuspended in injection buffer at a concentration 2 µg/µl.

## Supporting Information

Figure S1 **Alignment of blowfly TRA protein sequences.** Conserved domains 1 to 4 identified previously by [3] are represented in yellow, red, green and blue respectively. An arrow indicates the conserved exon-intron boundary within the putative auto regulation domain [11]. Arginine/serine-rich domains (RS domain) are shown in bold. Note that the LcTRA protein is slightly longer than originally reported due to a sequencing error, which has been corrected in the Genbank record.
(PDF)

Figure S2 **Alignment of blowfly** ***tra*** **male exon nucleotide sequences.** The TRA/TRA2 site is highlighted in red, the TACC/TA motif in blue and the T-rich motif in green. The first in frame stop codon is in bold.
(EPS)

Table S1 **MEME motifs in male exons.**
(PDF)

Table S2 **MEME motifs in male introns.**
(PDF)
