# Supplementary material for: Conservation and Sex-Specific Splicing of the transformer Gene in the Calliphorids Cochliomyia hominivorax, Cochliomyia macellaria and Lucilia sericata
Source: PLoS One. 2013 Feb 7;8(2):e56303. doi: 10.1371/journal.pone.0056303 (PMC3567074; doi:10.1371/journal.pone.0056303)
Supplement: Figure S1 — Alignment of blowfly TRA protein sequences. Conserved domains 1 to 4 identified previously by [3] are represented in yellow, red, green and blue respectively. An arrow indicates the conserved exon-intron boundary within the putative auto regulation domain [11]. Arginine/serine-rich domains (RS domain) are shown in bold. Note that the LcTRA protein is slightly longer than originally reported due to a sequencing error, which has been corrected in the Genbank record. (PDF) [file pone.0056303.s001.pdf]

```
Chtra     MDSNITGLSASSTLGGPKFKIHQSIPSGSVKRGPHAIVRSVELNDCIEIQRRCGEGSIPL
Cmtra     MDSNITGLSASSTLGGPKFKIHQSIPSGSVKRGPHAIVRSVELNDCIEIQRRCGEGSIPL
Lstra     MDSIATGLAASSTLEGTKFKIQQSIPSGSIKRGPHAIVRTADLNDGINIQRRFGEGSKPL
Lctra     MDSITTGLAASSILEGTKFKIQQSIPSGSIKRGPHAIVRTADLNDGINIQRRFGEGSKPL
          ***  ***:*** * *.****:*******:*********:.:*** *:**** **** **

Chtra     FDRHDITVNQTINESNDKQ-INHEHHTSGKT-KTNRRSSSRESSSPERYRHRDIKKHSST
Cmtra     FDRHDITVNQTINESNDKK-TNHEHHTGGKS-KTNRRSSSRESSSPERYRHRDIKKHSSS
Lstra     FDRDDIAVNQTTLDARDTNNLDHERNTGCKT-KTNRRSSSTVSSSPERYRHRDIKKHSS-
Lctra     FDRDDIAVNQTTLDARDTNNLDHERNTGCKTNKTNRRSSSTESSSPERYRHRDIKKHSS-
          ***.**:****  ::.*.:  :**::*. *: ********  *****************

Chtra     SMSRRRKTPERTVRSERPSNNRDHHSYNLKHG-SNTKTDKYSRKDKSKTRTPSPNK-KKP
Cmtra     SMSGRRKTPERTARSERPNNNRDHHSYNLKCSSSNTKTDKYRRKDRSKSRTPSPNK-KKP
Lstra     PTSGRRKTPERSGRNERPTHSHDKHNYNVKSN--NTMTDKYKRSRRSKSRSPPHNANKKT
Lctra     PTSGRRKTPERSGRSERPTHSHDKHNYNVKSN--NTMTDKYKRSRRSKSRSPPHNANKKT
          . * *******: *.***.:.:*:*.**:*  .  ** **** *. :**:*:*. *  **.

Chtra     GEKVPYYRDEQREKDRLKRLYGRSCSRSPPPTSRS-SGRKYSPITKRRIESP--RRRRSR
Cmtra     VEKVPYYRDEQREKDRLKRLHGRSCSRSPPPTSRS-SARKYSPATKRRIESPP-RRRRSR
Lstra     VEKVPYYRDEQREKDRIRRLYGRSRSRTPP------AAATLSSSTKRRIESPTRRRRRST
Lctra     VEKVPYYRDEQREKDRIRRLYGRSRSRTPPPPVGNLSSSSSSSTAKRRIESPSRRRRRST
           ***************::**:*** **:**      :. . *. :*******  *****

Chtra     SKDRYRKYSPH--RSSRKDYRTHRSSRSRSRSRTRSPSRRERHKHYSRSSRERDKEHKED
Cmtra     SRDRHRKYSPH--RSSRRDYRTHRSSRSRSRSRTRSPSRRERHKHYSRSSRERDKEHKED
Lstra     SRER-RRHSPY-HRNVRRDYR---SRRSRTRSRTRSPPRRERYKHSSRSSRERDNEHKED
Lctra     SRDRHRRHSPYIHRSVRRDYR---SRRSRTRSRTRSPQRRERHKHSSRSSRERDKEHKED
          *::* *::**:  *. *:***   * ***:******* ****:** ********:*****

Chtra     VNNRSTAILPPTPQFI-LPVAVPADY--AAAAYTFPGWTTAPQLAWHPGHHRPP---AAA
Cmtra     ANNRNTAILPPTPQFI-LPVAVPADYAAAAAAYSFPGWTAAPQLAWHPGHHRPPA--AAA
Lstra     ANSLTTAIIPATPQIIPIPVPVPAEY---AAAYTFPGWT-APQPTWPPSHPRPPSASHFA
Lctra     VNSLTTAIIPATPQIIPIPVPVPAEY---AAAYTFPGWT-APQPTWPPSHHRPPATSHFA
          .*. .***:*.***:* :**.***:*   ****:***** *** :* *.* ***     *

Chtra     PFPMVPMLPALRPPTHQASYAGLPP-ALAYPPITSPYRPHIPQRYPPPRHNTNN-YHSRP
Cmtra     PFPMVPMLPALRPPPHQASYAGLPP-ALAYPPITPPYRPHIPQRYPPPRHNTNS-YHSRP
Lstra     PFPMWTMMPPLRPPPHQASYGGLPPPALAYPPMAASYRPHAPQRYPQPRHDSNN-YQTRP
Lctra     PFPMWPMMPPLRPPPHQASYGGLPPPTLAYPPMTASYRPHGPQRYPQPRHNSNNNYQTRP
          **** .*:*.****.*****.**** :*****::..**** ***** ***::*. *::**

Chtra     KKPTS
Cmtra     KKPTS
Lstra     KKPSS
Lctra     KKPPS
          ***.*
```
