# Supplementary material for: Conservation and Sex-Specific Splicing of the transformer Gene in the Calliphorids Cochliomyia hominivorax, Cochliomyia macellaria and Lucilia sericata
Source: PLoS One. 2013 Feb 7;8(2):e56303. doi: 10.1371/journal.pone.0056303 (PMC3567074; doi:10.1371/journal.pone.0056303)
Supplement: Table S1 — MEME motifs in male exons. (PDF) [file pone.0056303.s003.pdf]

Table S1. MEME motifs in male exons

Motif 1

Species	Start	P-value	5' Flanking	MEME Motif	3' Flanking
Lc	230	5.55e-11	TATGAGACTT	CTTCAATCAACATACCATTT	TTTGGAGAAA
Ls	240	5.55e-11	GATGGGACTT	CTTCAATCAACATACCATTT	TTTTGGGAGA
Cm	224	1.18e-10	GAGGAGGAAA	CTACAATCAACATACTATTT	CTTGGTTTGA
Ch	220	1.87e-10	GAGGAACAAA	CTACAATCAACATACTATGT	CTTGGTTTGA

Motif 2

Species	Start	P-value	5' Flanking	MEME Motif	3' Flanking
Ch	30	2.35e-09	TCAAGTGTTA	CGGCTCCATTTTTCTTT	AGATATTTCC
Cm	30	5.59e-09	ACAAGAGTTA	TGGCTCCATTTTTCTTT	AGATATTTCC
Lc	29	1.41e-08	CCAAACCTAA	TTGCAGCAGATTTCTTT	TATTATTTCA
Ls	31	1.12e-07	AACCTTATTG	CTGCAGTAGTTTTCTTT	TCGTTTAGTT

Motif 3

Species	Start	P-value	5' Flanking	MEME Motif	3' Flanking
Lc	96	3.23e-08	ATTAGTCAAT	TTGGGATTTAGTTG	AAAAAAACTT
Ls	104	3.23e-08	ATTAGTCAAT	TTGGGATTTAGTTG	AAAAAAAAAG
Ch	109	1.01e-07	AAGCATACTG	TTGGGATTTAAATG	ATTTTTTTAT
Cm	111	1.86e-07	AAAAACATTC	TTGGGATCTAAATG	ATTTACTTTA

Motif 4

Species	Start	P-value	5' Flanking	MEME Motif	3' Flanking
Ch	269	2.44e-10	TGTGACATAT	TGGGCAGTTAAAACAAGACT	ATAAC
Cm	273	1.30e-09	TATGACGTAT	TGGGCAGTTAAGACAAGGCT	ATAAC
Lc	6	4.58e-08	GTAAT	TTTCCTTTTAAAACCAAACC	TAATTGCAGC
Ls	5	4.58e-08	GTAA	TTTTCCTTTAAAACCAAACC	TTATTGCTGC

Motif 5

Species	Start	P-value	5' Flanking	MEME Motif	3' Flanking
Ch	199	1.73e-08	TGAAAACGTT	GCATTTTCCAAGAGGA	ACAAACTACA
Ls	187	2.86e-08	TTGAGTACCA	TCAATTTCCTGATGGC	AAAGTGGAAA
Cm	203	3.99e-08	TGAAAACATT	GCATTCTCCAAGAGGA	GGAAACTACA
Lc	176	6.48e-08	TCGAATGGCA	TCAATTCCCTGATGGC	AAAGTGCATA

Motif 6

Species	Start	P-value	5' Flanking	MEME Motif	3' Flanking
Lc	265	4.10e-09	AGAAAACTAA	GGATGCAATCATTTGGCGG	TTAAAAAATC
Ls	274	6.61e-09	GGGAGACCAA	GGATACAATCAATTGGCGG	CAAAAGGCAA
Cm	252	3.97e-08	TTCTTGGTTT	GAGGAAGAACTTATGACGT	ATTGGGCAGT
Ch	248	1.69e-06	GTCTTGGTTT	GAAGAAGAAGTTGTGACAT	ATTGGGCAGT

Motif 7

Species	Start	P-value	5' Flanking	MEME Motif	3' Flanking
Ls	206	3.46e-09	TGATGGCAAA	GTGGAAAAATAGCTTGTCCT	TAAAGATGGG
Ch	170	4.57e-08	AGTTTTAATA	ATGGCAAAAGAAAATATACT	GAAAACGTTG
Lc	195	1.19e-07	TGATGGCAAA	GTGCATAAATAGCTTGTTCT	TGAAATATGA
Cm	174	5.39e-07	AGTTTTAATA	ATGACAAAAGAAAAAATGCT	GAAAACATTG

Motif 8

Species	Start	P-value	5' Flanking	MEME Motif	3' Flanking
Ls	49	1.18e-06	GTTTTCTTTT	CGTTTAGTTTGTT	TACAATATTC

Ch	57	1.74e-06	AGATATTTCC	AGTATAGTTTTTT	ATTACCAACA
Cm	57	3.38e-06	AGATATTTCC	AGTATAGTTCTTT	TATTATCAAC
Lc	54	1.04e-05	TTTATTATTT	CATTTAGTTTTTT	TATAATACAA

Motif 9

Species	Start	P-value	5' Flanking	MEME Motif	3' Flanking
Ch	136	1.10e-05	TTTTTATTAA	AAAGTGAGAC	AAAATTTTCA
Ls	228	1.33e-05	CTTGTCCTTA	AAGATGGGAC	TTCTTCAATC
Lc	218	1.60e-05	TTGTTCTTGA	AATATGAGAC	TTCTTCAATC
Cm	141	5.96e-05	TTTATTTAAA	AAAATGAAAC	ACATTTTGAA

Motif 10

Species	Start	P-value	5' Flanking	MEME Motif	3' Flanking
Lc	155	5.85e-09	GCTACTTTTA	AAGCTAAATATTCGAATGGC	ATCAATTCCC
Ls	166	1.30e-08	AACAACATTT	AAGCTAAATATTTGAGTACC	ATCAATTTCC
Cm	87	2.85e-06	AACAAGTTAA	AAGATAATTTTTTTAAAAAC	ATTCTTGGGA
Ch	84	7.08e-06	CCAACATTTA	AAAACAAATTTTAGAAAGCA	TACTGTTGGG

Motif 11

Species	Start	P-value	5' Flanking	MEME Motif	3' Flanking
Lc	82	8.00e-06	TACAATTAAA	ATTTATTAGTCAAT	TTGGGATTTA
Cm	153	8.00e-06	AATGAAACAC	ATTTTGAATACAGT	TTTAATAATG
Ls	90	8.00e-06	TACAATAACA	ATTTATTAGTCAAT	TTGGGATTTA
Ch	149	8.85e-06	GTGAGACAAA	ATTTTCAATACAGT	TTTAATAATG

Motif 12

Species	Start	P-value	5' Flanking	MEME Motif	3' Flanking
Ls	305	6.45e-06	AAAGGCAATA	AAGGCTCTTA	AGAG
Lc	299	3.54e-05	AAATCTTTAA	AGGGCCCTTA	AG
Cm	3	1.60e-04	GT	AATTTTCTTA	ACATATAACA
Ch	3	5.13e-04	GT	AATTTTCTTT	ACGTATATCA

Motif 13

Species	Start	P-value	5' Flanking	MEME Motif	3' Flanking
Ch	70	1.00e-05	ATAGTTTTTT	ATTACCAACA	TTTAAAAACA
Ls	74	4.37e-05	CAATATTCTA	TTTACCTACA	ATAACAATTT
Cm	71	7.37e-05	TAGTTCTTTT	ATTATCAACA	AGTTAAAAGA
Lc	141	3.45e-04	AAATGAAATG	ATAAGCTACT	TTTAAAGCTA

Motif 14

Species	Start	P-value	5' Flanking	MEME Motif	3' Flanking
Ls	264	2.29e-05	CCATTTTTTT	GGGAGACCAA	GGATACAATC
Cm	14	1.87e-04	ATTTTCTTAA	CATATAACAA	GAGTTATGGC
Ch	14	2.09e-04	ATTTTCTTTA	CGTATATCAA	GTGTTACGGC
Lc	254	3.45e-04	CCATTTTTTG	GAGAAAACTA	AGGATGCAAT

Motif 15

Species	Start	P-value	5' Flanking	MEME Motif	3' Flanking
Cm	47	3.10e-05	ATTTTTCTTT	AGATATTTCC	AGTATAGTTC
Ch	47	3.10e-05	ATTTTTCTTT	AGATATTTCC	AGTATAGTTT
Ls	131	2.16e-04	AAAAAAGTAC	AAAAAATTCA	TAAATTAAAA
Lc	122	3.70e-03	AAAAACTTAA	AAAAAATTAA	AATGAAATGA

Motif 16

Species	Start	P-value	5' Flanking	MEME Motif	3' Flanking
Cm	126	4.91e-04	ATCTAAATGA	TTTACTTTAT	TTAAAAAAAT
Lc	289	1.68e-03	GGCGGTTAAA	AAATCTTTAA	AGGGCCCTTA
Ls	154	7.20e-03	ATTAAAAAGT	TAAACAACAT	TTAAGCTAAA
Ch	123	1.45e-02	GATTTAAATG	ATTTTTTTAT	TAAAAAGTGA

Motif 17

Species	Start	P-value	5' Flanking	MEME Motif	3' Flanking
Cm	1	4.81e-04	.	GTAATTTTCT	TAACATATAA
Ch	1	4.81e-04	.	GTAATTTTCT	TTACGTATAT
Ls	293	1.18e-02	CAATTGGCGG	CAAAAGGCAA	TAAAGGCTCT
Lc	110	1.61e-02	GATTTAGTTG	AAAAAAACTT	AAAAAAAATT

Motif 18

Species	Start	P-value	5' Flanking	MEME Motif	3' Flanking
Cm	1	2.57e-04	.	GTAATTTTCT	TAACATATAA
Ch	1	2.57e-04	.	GTAATTTTCT	TTACGTATAT
Ls	62	9.91e-03	TTAGTTTGTT	TACAATATTC	TATTTACCTA
Lc	72	1.20e-02	TTTTTTATAA	TACAATTAAA	ATTTATTAGT

Motif 19

Species	Start	P-value	5' Flanking	MEME Motif	3' Flanking
Cm	1	2.64e-05	.	GTAATTTTCT	TAACATATAA
Ch	1	2.64e-05	.	GTAATTTTCT	TTACGTATAT
Lc	1	2.94e-05	.	GTAATTTTCC	TTTTAAAACC
Ls	118	1.58e-01	GATTTAGTTG	AAAAAAAAAG	TACAAAAAAT

Motif 20

Species	Start	P-value	5' Flanking	MEME Motif	3' Flanking
Cm	1	2.34e-05	.	GTAATTTTCT	TAACATATAA
Ch	1	2.34e-05	.	GTAATTTTCT	TTACGTATAT
Lc	1	2.84e-05	.	GTAATTTTCC	TTTTAAAACC
Ls	142	1.96e-01	AAAAATTCAT	AAATTAAAAA	GTTAAACAAC
