# Supplementary material for: Conservation and Sex-Specific Splicing of the transformer Gene in the Calliphorids Cochliomyia hominivorax, Cochliomyia macellaria and Lucilia sericata
Source: PLoS One. 2013 Feb 7;8(2):e56303. doi: 10.1371/journal.pone.0056303 (PMC3567074; doi:10.1371/journal.pone.0056303)
Supplement: Table S2 — MEME motifs in male introns. (PDF) [file pone.0056303.s004.pdf]

Table S2. MEME motifs in male introns.

Motif 1

Species	Start	P-value	5' Flanking	MEME Motif	3' Flanking
Lc	1997	3.06e-09	AAATGTTTCT	CTCAACAATCAACAT	ACCAAAATTG
Cm	2431	3.06e-09	AAAAATAAAA	CTCAACAATCAACAT	ACCAAAATTT
Ls	1619	3.06e-09	AAATGTTCCT	CTCAACAATCAACAT	ACCAAAATTG
Ch	2092	3.06e-09	ATAATAAAAG	CTCAACAATCAACAT	ACTAACATTT

Motif 2

Species	Start	P-value	5' Flanking	MEME Motif	3' Flanking
Lc	1788	3.18e-09	TAAAACAATC	AACAATCAACATACC	AAAGTTTTGA
Cm	2631	3.18e-09	AAAAATCAAC	AACAATCAACATACC	ATAATTGAAA
Ls	1418	3.18e-09	TAAAACAATC	AACAATCAACATACC	AAATTTTGAA
Ch	2290	3.18e-09	TAAAAATAAC	AACAATCAACATACC	ATAATTGAAG

Motif 3

Species	Start	P-value	5' Flanking	MEME Motif	3' Flanking
Ch	1094	1.41e-10	TCTCTACTGG	CCTTAGCTCCGGGC	TTTTTGGAGA
Lc	1013	2.05e-09	CAGATGTAAA	CCTTACCTCTGGGA	GAGTATTCAG
Cm	1876	4.58e-09	CTCTCCACAG	CCTTAACTCTGGGC	AGTGGAATAT
Ls	828	7.90e-08	ACTGAATTAT	CCATGCCTGCCGTA	CTATCTATCT

Motif 4

Species	Start	P-value	5' Flanking	MEME Motif	3' Flanking
Ch	2160	1.10e-09	TTATAATTAA	CGACAATCAACATAC	CATAGAAAAA
Lc	1864	4.28e-09	TTCAATTAAA	CAACAATCAACATAC	TATTTAATGA
Ls	1490	4.28e-09	TTTTCCTAAA	CAACAATCAACATAC	TATTTATTAA
Cm	2500	7.35e-09	CTATAATTAT	CTACAATCAACATAC	CAAATAAAAA

Motif 5

Species	Start	P-value	5' Flanking	MEME Motif	3' Flanking
Lc	1908	7.29e-09	CGTCAACAAA	ACTACAATCAACATT	ACCTTAACTC
Cm	2551	7.29e-09	TGAACACCAA	ACTACAATCAACATT	TCCCTCAAGG
Ls	1534	7.29e-09	CATCAACAAA	ACTACAATCAACATT	ACCTAAACCC
Ch	2209	7.29e-09	TGAATACAAA	ACTACAATCAACATT	TTCCTCAGGG

Motif 6

Species	Start	P-value	5' Flanking	MEME Motif	3' Flanking
Ch	2244	7.09e-10	CAACACACAT	CTAGGTTTTGCAAGG	ATCAACAAAT
Lc	220	1.57e-09	CCAGCACAAA	CTGGTTTTTGTAAGG	AATATAGACT
Cm	2584	3.95e-09	GGCAACACAC	CAAGGTTTTGCAAGG	ATCAACAAAC
Ls	855	1.96e-08	TCTATCTAAA	CTGATTTTCGTAAGG	AATATCGACT

Motif 7

Species	Start	P-value	5' Flanking	MEME Motif	3' Flanking
Cm	4022	7.23e-08	TAATTTAAAA	GGATCCAGGG	TTACATGTAT
Lc	2353	2.01e-07	GAATATGTTT	GTTTCCAGGG	ATAATTATTA
Ls	2014	2.01e-07	AAATATGTTT	GTTTCCAGGG	ATAATTATTA
Ch	2631	1.44e-06	GATTTTAAAA	GGATCGAGGT	TGCATGTATG

Motif 8

Species	Start	P-value	5' Flanking	MEME Motif	3' Flanking
Lc	2321	6.85e-09	TTATTATCAA	CTTTAGCAACATAC	AAAAAAATGA

Ls	1985	6.85e-09	TTATTATCAA	CTTTAGCAACATAC	GAAAAAAATA
Cm	3983	2.59e-08	AATTAATCAT	CTTTATCAACATAC	AAAATGATAG
Ch	2594	2.59e-08	AATTAATCAT	CTTTATCAACATAC	AAAATGATAG

Motif 9

Species	Start	P-value	5' Flanking	MEME Motif	3' Flanking
Ls	1563	5.40e-09	AAACCCAACA	GCAACATGGCAACA	AAATACAAAA
Ch	2225	5.76e-09	ATCAACATTT	TCCTCAGGGCAACA	CACATCTAGG
Cm	2567	7.33e-09	ATCAACATTT	CCCTCAAGGCAACA	CACCAAGGTT
Lc	1937	3.79e-08	TAACTCGGCA	GCAATATGGCAACA	AAAAGTACAA

Motif 10

Species	Start	P-value	5' Flanking	MEME Motif	3' Flanking
Lc	1758	4.28e-09	GTTTTAAGTA	TAATGAATTTCAGTG	AAATATAAAA
Cm	2406	4.28e-09	TAGTTAAGTT	TAATGAATTTCAGTG	AAAAATAAAA
Ch	2066	4.28e-09	TAGTTAAGAT	TAATGAATTTCAGTG	AATAATAAAA
Ls	1388	3.26e-08	AAGTTAAGTA	TAATGATTTTCAGTA	AAATATAAAA

Motif 11

Species	Start	P-value	5' Flanking	MEME Motif	3' Flanking
Ch	926	3.66e-08	CGTTCTTCGA	GGGGCGGTCA	TAATATTTTT
Cm	470	3.06e-07	TTCTAGAGAT	GGCGCAGTCA	ATAACGAGAC
Lc	1134	8.40e-07	ATTAAGCGCT	GTGGGCGCCA	TATTTCTTGC
Ls	985	1.08e-06	GACTGACGTT	GTCGGAGTCA	ATTCACGTTG

Motif 12

Species	Start	P-value	5' Flanking	MEME Motif	3' Flanking
Cm	380	2.28e-10	CATAGATCTT	CTGGAGTAAGAGTGG	TAGCTTCGCT
Ch	223	2.28e-10	CCTGGATCTT	CTGGAGTAAGAGTGG	TAGGATTGGC
Lc	732	4.29e-08	CATAAATGTG	CAGGAATCAGAGTAC	GTAATTCTAA
Ls	757	7.72e-08	TAAGTTAGAA	ATGTCGTGAGAGTTC	CACGATATTT

Motif 13

Species	Start	P-value	5' Flanking	MEME Motif	3' Flanking
Cm	517	6.70e-10	GCCTCTATCA	CGAGTGTAGTGTGC	TAGAACTACC
Lc	290	5.28e-09	ACATCAAATC	CGCCTGTTGTGCGC	TCCTTATCAT
Ls	875	7.87e-08	TAAGGAATAT	CGACTGAAGTCGGT	AGAATATTCA
Ch	325	1.08e-07	ACGGTCAATA	CGAGACTAGTATGC	ATCGAGACTA

Motif 14

Species	Start	P-value	5' Flanking	MEME Motif	3' Flanking
Lc	1807	1.85e-09	CATACCAAAG	TTTTGAAGACCAGGG	TAAAGGTTTC
Cm	2453	5.65e-09	CATACCAAAA	TTTTGAAGATCAGCA	ATATTAATCT
Ch	2114	5.65e-09	CATACTAACA	TTTTGAAGATCAGCA	ATATTAATCT
Ls	1436	4.32e-08	ACATACCAAA	TTTTGAAGATTCCGG	CAAAGGTTTT

Motif 15

Species	Start	P-value	5' Flanking	MEME Motif	3' Flanking
Lc	1892	1.42e-08	TTAATGAAAT	GGACAACGTCAACA	AAACTACAAT
Ls	1518	1.42e-08	TTATTAAAAT	GGACAACATCAACA	AAACTACAAT
Ch	308	6.93e-08	CAAATATTAG	GGACGGCACGGTCA	ATACGAGACT
Cm	2824	1.50e-07	TAGTAGAGAA	GGAAATCATCCACA	TAAAAAAATT

Motif 16

Species	Start	P-value	5' Flanking	MEME Motif	3' Flanking
Lc	520	1.04e-07	TACATCTGAA	TCCGCGCGTC	CAATTTTGCA
Ch	705	2.06e-07	AGAAAAATAG	TCCGCTCCCC	TACTTGATGC
Ls	310	1.55e-06	AAACAAATTT	TCCGCTAGAC	TATAGATACA
Cm	1022	4.69e-06	ATGTAGTTTT	TCTGCTCGTT	CTTCAAGCCA

Motif 17

Species	Start	P-value	5' Flanking	MEME Motif	3' Flanking
Ch	732	2.46e-10	TGCCCATATT	CGCATTCGGTGTAG	TCATCTTGTT
Cm	973	3.13e-08	TGTCCATGTT	CACATTCAGTGTCG	CAATTTTCCA
Lc	1368	5.83e-08	AATTCACTTA	CTGATTCCGTGTAG	TTTTGTATAA
Ls	1025	3.49e-07	TAACACATAA	GACACCCGCTATAG	TGTTGAATTT

Motif 18

Species	Start	P-value	5' Flanking	MEME Motif	3' Flanking
Ch	555	4.60e-08	TTGATATAAA	CCTTATTCCAGGG	TACTAAAGAT
Lc	1687	6.14e-08	TTTAAATCTT	CCTATTTCAATGG	AATAGAAAAA
Ls	1323	6.14e-08	GTTAAATCTT	CCTATTTCAATGG	AATACAAAAA
Cm	798	3.17e-07	TTGATATAAA	TCTAATTCCAAGG	TACTGAAGAA

Motif 19

Species	Start	P-value	5' Flanking	MEME Motif	3' Flanking
Cm	3336	9.42e-08	AACGACAGAC	AGGGAGGCGG	TTATACGAAT
Lc	450	1.08e-06	CTTTGACGAA	TGCCTGGCTG	TGGCTAAGAA
Ch	1630	1.08e-06	CATAGGAAAC	AGCTTGGCGG	CAAACCTTTG
Ls	1590	8.41e-06	TACAAAAGTA	TGGAAGTCTG	AATGAACGAA

Motif 20

Species	Start	P-value	5' Flanking	MEME Motif	3' Flanking
Ch	1507	2.47e-07	GACCAATGAG	CTAACCGGGC	TTTTCTACCT
Cm	3032	9.01e-07	TCAAAGATTC	CTAACCCAGC	CACTTAAAAT
Ls	200	9.01e-07	GGAAAAAGTA	TTAACCCGCC	GATTAAGTAT
Lc	1926	1.29e-06	CAACATTACC	TTAACTCGGC	AGCAATATGG
